# 817 New Mitophagy Assays to Investigate Deficient Mitophagy Responses in Burn Injury: Implications for Translational Research

**DOI:** 10.1093/jbcr/iraf019.348

**Published:** 2025-04-01

**Authors:** Yoh Sugawara, Hiroki Ogata, Hiroyuki Morinaga, jingyuan Chen, Jeevendra Martyn, Shingo Yasuhara

**Affiliations:** Shriners Children’s - Boston; Shriners Children’s - Boston; Shriners Children’s - Boston; Shriners Children’s - Boston; Shriners Children’s - Boston; Shriners Children’s - Boston

## Abstract

**Introduction:**

Autophagy and mitophagy are essential cellular quality control mechanisms, with their modulation presenting a promising therapeutic strategy for critical illnesses. We previously developed a mitophagy assay using the Kaede reporter, but sought to eliminate the reliance on transgenes. By integrating MitoTracker dye with a bioengineered microplate for real-time monitoring, we established a method to measure mitophagy in non-fluorescent samples.

**Methods:**

We developed a method to monitor mitophagy flux without transgene reporters by screening mitochondrial dyes that remain stable after CCCP-induced damage. Using this dye, we tracked mitophagy in non-fluorescent wild-type cells and validated it against Parkin KO cells and mitophagy inhibitors. To conduct chronological cell tracking before and after cellular stimulation, we constructed a real-time fluid exchange system and combined it with this assay. The assay was applied to naive (non-fluorescent) cells freshly harvested from severe burn injury mice, showing decreased mitophagy flux. This method offers a robust tool for studying mitophagy in non-fluorescent cells and primary samples.

**Results:**

Unlike most mitochondrial dyes that lose fluorescence after membrane potential loss, MitoTracker allowed extended observation post-mitophagy activation. It enabled accurate quantification of mitophagy flux by measuring fluorescence decline, indicating mitochondrial degradation (66.6% reduction). In Parkin knockout cells and with mitophagy inhibitors, signal decay was significantly blocked (14.9% reduction). In burn-injured mouse macrophages, mitophagy response was impaired (Sham: 51.9% vs. Burn: 22.4%).

**Conclusions:**

We developed a quantitative assay for mitophagy on a real-time fluid exchange chamber, utilizing non-fluorescent naive cell lines and primary macrophages isolated from mice, setting the stage for future use with human patient samples. This innovative assay successfully detected impaired mitophagy in models of burn injury. Notably, it represents a significant advancement in evaluating mitophagy function in live non-fluorescent samples, creating opportunities for mechanistic research and guiding personalized therapeutic strategies aimed at correcting dysregulated mitophagy in clinical settings.

**Applicability of Research to Practice:**

The new mitophagy assay can be applied to human samples, enabling real-time analysis of mitophagy in patient-derived cells without genetic modification. Clinically, this could identify patients with burn injuries or other critical conditions who have impaired mitophagy, allowing for personalized treatments. Additionally, the assay may support drug screening for novel therapies, enhancing recovery outcomes through targeted interventions.

**Funding for the Study:**

Shriners Hospitals for Children Research Grant (#85106)

Hyogo Medical University Fellowship

